# 1104. Case Study. Successful Split-thickness Skin Graft on a Gouty Tophus: A Case Report

**DOI:** 10.1093/jbcr/irag033.208

**Published:** 2026-04-06

**Authors:** Judah Hoffman, Catherine Toal, Rachelle J Lodescar

**Affiliations:** Burn Center at Westchester Medical Center, NY, New York; Burn Center at Westchester Medical Center, White Plains, NY; Westchester Medical Center, Valhalla, NY

## Abstract

**Patient Presentation (age range, injury details, relevant history):**

A 66-year-old man with a history of gout and severe right foot burns was transferred from a local hospital after suffering injuries while attempting to stomp out a fire that had engulfed his shirt. The patient sustained 1.25% total body surface area full-thickness circumferential burns to the digits of the right foot and multiple areas of blistering burns to the left foot (Fig. 1). Upon arrival at the hospital, the patient received local wound care and intravenous antibiotics for cellulitis. On post-burn day 8, after improvement in swelling and infection, tangential excision and extracellular matrix placement were performed on the left foot with good graft take.

**Clinical Challenges:**

On post-burn day 20, the patient underwent repeat operative management and the allograft was excised. A significant tophaceous growth was observed and excised over the proximal interphalangeal joint of the second digit of the right foot. Once adequate vascular supply for grafting was achieved, excision was stopped due to concern for damage to joint structures and risk of joint infection. In severe cases of tophaceous gout, surgical excision is often indicated to restore joint function. However, in this case, the tophaceous crystals were interspersed with important joint structures, rendering complete surgical excision challenging.

**Management Approach:**

After careful evaluation, the decision was made to prioritize wound closure and infection prevention over removal of the tophus. To avoid amputation of the digits, the graft was applied directly over the tophus, the right foot, and right digits one through four, using a 1:1 meshed split-thickness skin autograft harvested from the right thigh.

**Outcomes:**

At discharge 7 days after the second operation, and again at a follow-up appointment one week after discharge, the graft demonstrated appropriate vascularization and adherence, including over the gouty tophus (Fig. 2).

**Lessons Learned:**

In medically complex patients such as this one, complete surgical excision with possible amputation of the digits may carry greater risk than benefit. This case study suggests that autografting over a gouty tophus has the potential to successfully adhere and heal when complete excision is not feasible.

**Applicability to Practice:**

Successful grafting over a gouty tophus has not been previously reported in the literature. Given the increasing prevalence of gout, this case may serve as a guide for the viability of skin grafting onto gouty tophi. Further studies are needed to optimize treatment and guidelines for this rare indication.

